# Wastewater surveillance in the military: how deployed members of the armed forces can monitor outbreaks on military vessels

**DOI:** 10.3389/fepid.2025.1630930

**Published:** 2026-01-14

**Authors:** Anna Gitter, Kristina D. Mena, Michelle Crum, Erick Butler

**Affiliations:** 1 Department of Environmental and Occupational Health Sciences, School of Public Health University of Texas Health Science Center at Houston, El Paso, TX, United States; 2 Department of Preventive, Occupational, and Environmental Medicine, The University of Texas Health Science Center at Tyler, Tyler, TX, United States; 3 College of Engineering, West Texas A&M University, Canyon, TX, United States

**Keywords:** pathogens, microbiology, SARS-CoV-2, wastewater, low-income and middle-incomecountries, colleges and universities, disease monitoring

## Abstract

This perspective piece explores the potential to implement wastewater surveillance on military vessels to improve disease monitoring and prevention. We examine five key topics: (1) recent studies of wastewater surveillance on military bases and training centers; (2) best practices for confined populations (e.g., colleges, prisons, hospitals, and low-income and middle-income countries) and their transferability to military settings; (3) current technologies enabling deployed personnel to conduct wastewater surveillance without advanced microbiological training; (4) key questions the military should address to prevent future outbreaks on vessels; and (5) unique ethical considerations surrounding implementation. This work aims to inform military decision-makers considering the adoption of wastewater surveillance programs.

## 1 Introduction

“We are not at war. Sailors do not need to die.”

-Captain Brett Crozier, commander of the U.S.S. Theodore Roosevelt during the COVID-19 pandemic (1).

During the early months of the COVID-19 pandemic, a viral outbreak occurred on the U.S.S. Theodore Roosevelt while the ship was out at sea in the Western Pacific (2, 3). During this time, more than 1,300 crew members were infected with the SARS-CoV-2 virus, with 1,271 testing positive and 60 suspected without a positive test. Consequently, this resulted in the re-routing of the vessel to the Naval Base in Guam, where crew members were isolated, hospitalized, or quarantined in hotel rooms or at the Naval Base. In total, 69% of the crew members who tested positive were under 30, 23 were hospitalized, 4 received intensive care, and one died. It was suggested that crew members were more likely to be hospitalized due to pre-existing conditions. The aftermath of the incident resulted in the removal of several key officials involved with the outbreak. The lessons learned from this incident demonstrate the critical importance of increasing awareness and proficiencies when shipboard for United States (US) armed forces to prepare and mitigate infectious disease outbreaks (2, 3). Interestingly, at the time of the localized outbreak, it was discovered that medical personnel onboard were only prepared to handle first aid situations and not infectious diseases (3).

Prior to the outbreak, the Under Secretary of Defense for Personnel and Readiness (USD(P&R)) prepared documentation detailing how personnel within the Department of Defense (DoD) should handle situations concerning the SARS-CoV-2 Virus. Titled “Consolidated Department of Defense Coronavirus Disease 2019 Force Health Protection Guidance,” this manuscript was originally prepared from the perspective of not allowing a public health emergency to prevent the execution of tasks by the armed forces. On January 30, 2023, an update was made to the chapter associated with surveillance and screening testing. In this chapter the USD(P&R) recommends the “leveraging…wastewater surveillance to supplement existing COVID-19 surveillance systems  (4)”. The use of wastewater surveillance by the armed forces has been documented in only five published studies (5–9), with three studies (5–7) involving the US Air Force, and one including the Coast Guard (8), and one with the French navy (9). Given the sensitive nature of military operations, limited information on additional programmatic implementation is unavailable. A non-systematic review of the publicly available studies is as follows.

1. The United States Air Force Academy (USAFA) in Colorado Springs, CO implemented a wastewater surveillance program for SARS-CoV-2 from influent wastewater from the local wastewater treatment plant. The results from this tempo-spatial study demonstrated the feasibility of surveillance by air force personnel (5).
2. A cost-analysis was performed to estimate the difference in costs of implementing surveillance on all US air force bases in contrast with clinical based testing. The authors concluded that wastewater surveillance would cost $10.5–$18.5 million less than clinical testing (6).
3. Several authors assessed a system known as VirCapSeq-VERT, a metagenomics strategy, to identify varying genetic material within wastewater samples from the US Air Force Academy. During a biweekly sampling campaign for 13 months (February 2022 to May 2023), the authors were able to detect 68 families of viruses (7).
4. Researchers led a pilot study at three different US Coast Guard training centers and directly on four docked cutter vessels. The results from these studies concluded that testing is viable at offshore training centers, but exhibited some important considerations if sampling and analysis on cutters were to be performed (8).
5. The Institute for Biomedical Research of the Armed Forces in partnership with several other French institutions established two monitoring campaigns for SARS-CoV-2 directly on the aircraft carrier PA Charles-de-Gaulle. These occurred during the alpha and delta waves. The results from this study enabled swift decisions shipboard to mitigate viral spread. In addition, this study emphasizes the need for methods that can be applied by non-experts (9).

In summary, wastewater surveillance can be beneficial and prudent in its execution within the armed forces branches, therefore aiding the military in preparing for a potential outbreak. However, there are several opportunities for expanding these programs into areas such as directly on seaborne vessels. This piece discusses the considerations for further expansion of wastewater surveillance within the military. This is a perspective piece that is divided into three focus areas—public health considerations for military personnel, advancements in technology, and preparations for the next pandemic. For simplicity, we will focus our attention on seaborne vessels, such as naval ships. Please note that we use the words “vessel” and “ship” interchangeably throughout this piece. The word “aircraft vessel” refers to a specific type of vessel or ship. We will define this type of ship in an upcoming section. It is also important to note that this piece does not include any primary data collection, as this is limited to published studies, unpublished information from military websites, and experience with wastewater surveillance within other applications. While one of the authors has had some experience working with an armed forces branch on wastewater surveillance, none of the authors have done any on-site wastewater surveillance testing.

## 2 Public health considerations for military personnel

While there are at least three different studies assessing the performance of wastewater surveillance within armed forces (5–9), there are other populations or scenarios that could benefit from similar wastewater programs. Military branches can, in theory, mirror settings like civilian confined population groups (e.g., colleges and universities, nursing homes/long-term care facilities, hospitals, incarcerated populations at correctional facilities, and low-income and middle-income countries). In this section, we will briefly discuss why these specific populations are useful examples to glean implementation and management strategies for wastewater surveillance in military applications.

Prior to discussing the similarities between other settings, we would like to provide a brief overview of operations on a naval ship. Understanding naval ship operations is critical to identifying the best strategies in employing a regular wastewater surveillance program shipboard. The size of a navy ship can vary based on the type and size of the ship. Each ship is built to serve a different function, which can include aircraft support, combat, hospital services, and transport (10). Consider aircraft carriers like the *USS Theodore Roosevelt*. These massive ships are equipped with an airstrip and hangar to support aircraft management and storage. There are approximately 50 aircraft carriers in the world, with the US Navy possessing 11 of those 50 carriers (11). In general, an aircraft carrier is designed to support a crew with a maximum capacity between 5,000 and 6,000 members (12, 13). According to the US Navy website, crew deployment can last for 6–9 months (14). Depending on the size of the ship and the crew, it is possible for crew members to have difficulty navigating from one point of the ship, as walking spaces can be narrow (2). The limited space on the *USS Theodore Roosevelt* posed a challenge during the COVID-19 outbreak, as it enabled viral spread throughout the ship (2).

Colleges and universities—wastewater surveillance was broadly implemented during the COVID-19 pandemic at over 250 higher education institutions (15). Developing a strategy to monitor wastewater within military settings can be likened to efforts on university campuses for several reasons. Similar to military vessels, university campuses consist of younger populations. Research from these institutions offer valuable insights into effectively deploying younger individuals to collect and test wastewater samples for infectious diseases. Furthermore, the residential settings at these universities, which sometimes resemble the communal living arrangements on military bases and within military vessels, allow opportunities to adapt research strategies to facilitate optimal data collection strategies in those environments. Monitoring at institutions emphasizes the focus on developing on-site testing programs that provide timely results (16). College and university monitoring observations suggest that if an active clinical testing program for analytes already exists, the evaluation of wastewater monitoring may be considered effective, especially when evaluating the granularity of data directly with clinical data. This could be more representative, accurate, and timely than relying on wastewater monitoring results from a nearby city (17).

Nursing homes, long-term care facilities, and hospitals—nursing homes and long-term care facilities are a different type of residential living that would be beneficial for assessment. Clinical testing should be employed to corroborate the results of a wastewater monitoring program. Residents of nursing homes and long-care facilities are at risk of having high morbidity and mortality rates (18–21). Therefore, a wastewater monitoring program with high sensitivity and timeliness is important. In addition, if applicable, antiviral treatment may be recommended for those in these facilities based on their age and risk factors. Wastewater monitoring can provide an early alert to an outbreak that can then be followed by individual clinical testing to prevent increased morbidity and mortability in these settings for residents. Another population to consider is in some of these facilities include two distinct populations—employees and residents. These two population groups can be analyzed for parameters such as viral shedding and positivity rates (20). The transitive nature of employees and residents at nursing homes and long-term care facilities, particularly their similarity to military deployments (where individuals temporarily relocate and then return), also has implications for wastewater analysis, such as diversifying the composition (e.g., pathogen loading, volume) of wastewater (18, 21). Finally, reliance on incontinence products (such as underwear) by some residents may lead to missed test results, potentially obscuring the true extent of infection and hindering the identification of viral transmission within the nursing home (20, 21).

Hospitals have similar populations to nursing homes and long-term care facilities in that the populations are often immunocompromised and susceptible to infectious disease transmission. Therefore, it is imperative that a wastewater surveillance system is utilized effectively to provide appropriate warning when cases are increasing. Hospitals are high-resource environments, though, with personnel, advanced technologies, and medical care available for the prevention and response of disease transmission [see transmission (22)]. Additionally, hospitals are ideal settings for evaluating the correlation between virus signals in wastewater and clinical cases (23). Wastewater surveillance at military bases will likely differ from this implementation. For example, military bases and have a younger population, affecting viral shedding rates (24). Also, laboratory space and resources are limited, which will necessitate different methods for testing and surveillance. A real-time wastewater surveillance system could be integrated into a military workflow given the research and experience gained in hospital settings can offer valuable insights into the potential of such surveillance, particularly given the typically robust resources available to hospital surveillance programs.

Incarcerated populations at correctional facilities—although correctional facilities and military bases both offer a degree of environmental control due to their confined populations; they also present unique challenges for wastewater surveillance implementation. First, each correctional facility has different rules regarding visitor and staff movement and analyte screening (25). Second, different correctional facilities will have to use different wastewater sample collection points to ensure accurate detection of targeted analysis, due to differences in infrastructure and facility layout (26). In contrast to those challenges, it is possible to find a correlation between clinical cases and the wastewater signal provided sufficient administration of tests (25). Wastewater monitoring in prisons could also provide valuable insight due to the potentially broader range of detectable analytes such as prescription and recreational drugs that can be moved into and throughout a facility (25, 26) compared to community wastewater surveillance. This is important for military applications, as it provides a basis for selecting appropriate target analytes (25, 27). Morbidity and mortality rates are higher in prison than at community-levels (25, 27), indicating the dire need for robust surveillance programs to monitor and mitigate disease transmission.

Low-income and middle-income countries (LIMCs)—best practices from surveillance within LMICs can be beneficial primarily because many communities still employ decentralized wastewater treatment (28–31). This means that monitoring is not limited to the collection of wastewater from sewer infrastructure, but also alternative sources such as surface water, given the presence of sewage within them, due to insufficient treatment and discharge practices (29–31). Like the military applications, knowing that wastewater must be collected from different sources (deployment and on bases) to best identify and mitigate viral spread is also important within the context of LMICs. In addition, surveillance programs are typically limited by human and financial capital (28, 30), meaning that methods of sampling must be simple and effective. Also, sampling techniques for collection are not limited to grab and automatic samplers, as passive sampling would be a viable option if surveillance were to commence in these areas (31).

All of these captive population settings provide examples and applications that can be employed for military operations and by military personnel on vessels. First, wastewater surveillance programs that focus on younger populations have already been implemented at various scales across colleges and universities (15–17). The ability to not only conduct the sampling but also employ young individuals with limited training to assist with these programs has been previously highlighted (15–17). Similarly, while these programs initially started with SARS-CoV-2, they have continued with additional analytes, including influenza. Nursing homes and hospitals generally serve older populations (e.g., geriatric), but the transitory nature of these facilities highlights the unique sewage conditions that exist at these facilities. Military personnel may deploy and return, which is like the nature of patients at both hospitals and nursing homes. Additionally, these facilities serve immunocompromised or high-risk individuals, therefore emphasizing the critical need for sensitive and timely sampling and analysis practices to mitigate morbidity and mortality, which is a similar goal for military operations wanting to maintain personnel readiness. Lastly, incarcerated populations at correctional facilities and LMICs highlight the limited resources and training that often exist, yet the ability to employ innovative approaches to implement these wastewater surveillance programs. Identifying alternative sampling sources besides that of wastewater (such as surface water) showcases how each environmental setting warrants unique considerations. Additionally, certain equipment and resources may not be available. Therefore, passive sampling approaches or methods that rely on limited skills should not be disregarded but rather reexamined for innovative approaches.

Best practices for wastewater analysis on naval ships can be informed by what has been conducted in other closed settings, and also by real-time, broader public health surveillance. The suspicion or documentation of an acute, infectious disease epidemic on land may warrant a tailored, rapid response to wastewater sample collection and analysis on a vessel; however, naval ships could also implement a monitoring program to be used to routinely predict vessel crew health, anticipate medication inventories on board, and plan disease transmission mitigation. Wastewater samples should be collected prior to any treatment application, and optimally include collection from ports from blackwater holding chambers and/or wastewater lines coming from lavatories (Table 1). The design of a wastewater monitoring regimen on a naval vessel would initially involve the identification of ports for composite sample collection at time intervals that are consistent yet practical and economically feasible for the crew. The specific sampling interval may be defined based on voyage duration and incubation periods of infectious agents of interest. It is anticipated that sampling would be at least weekly but preferably twice per week, as well as align with vessel port stops. Negative and positive controls should be run with each analysis. Community-wide public health data regarding infectious disease incidence and prevalence, seasonal disease trends, and available vessel crew health data should be considered in determining hazardous agents to target in the analysis.

**Table 1:** Considerations for wastewater monitoring best practices for naval ships.

Factor	Example of best practice
Sample location	Blackwater chambers; wastewater lines
Sample type	Composite samples preferably; grab samples in acute situations
Frequency of sampling	Twice per week, if possible; more frequently based on voyage duration and suspected outbreak
Types of analysis	Microbial agent of interest based on disease trends, suspected outbreak

## 3 Advancements in technologies

Military personnel tasked with collecting data for wastewater monitoring of biological constituents typically lack specialized training in microbiology. However, this knowledge gap offers an opportunity to train crew members on basic methods, much like citizen science. The phrase “citizen science” was not coined until the 1990s (32), has become a staple in a variety of research contexts that range from bird watching to water and air quality monitoring (33). Most notably, citizen science efforts now involve utilizing complex instruments (e.g., global positioning system receivers (GPS) and motion-rigged cameras) for data collection (32). For example, amateur scientists were trained to use a portable qPCR machine to measure DNA in water samples (32). This study exemplifies the potential for similar training of military personnel for wastewater surveillance.

Since the COVID-19 pandemic, various companies have manufactured and made available portable qPCRs in an effort to make monitoring analytes within wastewater simpler. The cost of these instruments range from $500 to $20,000, with the ability to use either proprietary or third-party kits. These instruments can make it possible for sewage monitoring and data interpretation to be accessible and feasible by any personnel regardless of microbiological expertise. And while these instruments make this work possible, it is important to consider the challenges associated with citizen science efforts. These include the importance of proper positive and negative controls, establishment of a limit of detection based on the methodology utilized, and ensuring quality control by an initial validation of the methodology, followed by ensuring data accuracy by using duplicate or triplicate sample processing. Also, training and personnel expertise on the specific protocols and maintaining consistency in data interpretation, and quality assurance and control are crucial components to any wastewater interpretation to be effective at prevention (32–36). The limit of detection also varies between instruments, significantly impacting the ability to detect viruses at specific prevalence rates (34). Anecdotally, the Water Environmental Federation (WEF) and the Hach Company partnered with several correctional facilities to monitor COVID-19 within wastewater. In this pilot study, volunteers from the facilities were trained on how to collect wastewater samples at the facility and analyze them using a portable qPCR with its proprietary test kit to estimate the concentration of the SARS-CoV-2 Virus within their facility's wastewater (36). A key finding of this study is the lack of confidence among sewage monitoring participants regarding the accuracy of results. For example, despite a confirmed COVID-19 outbreak within a facility, wastewater testing produced contradictory results (36).

Additionally, to interpret trends in wastewater data, the following approaches are essential: first, ensure viral concentrations are above the limit of detection to confirm reliable presence. Then, normalize the data to account for variations in flow, population, and fecal strength. Analyzing temporal patterns—such as increases, decreases, or plateaus—can reveal shifts in community transmission, especially when smoothed with moving averages. Comparing wastewater trends with clinical indicators like case counts (or emergency room visits) and hospitalizations help contextualize findings, as wastewater often provides early signals. Finally, consider external factors like rainfall, testing behavior, and variant characteristics, and use statistical tools to assess the significance and reliability of observed changes. While these methods are utilized for analysis of wastewater data, those in a confined area such as a vessel or within a small community like a nursing home or correctional facility will be a microcosm of the typical larger community. Therefore, aspects of monitoring infections in populations may behave differently from normal patterns, and indicators like increases to the emergency room for certain infections may not be seen depending on the acceptability of reporting illness and probability of medical treatment being available for individuals. This could especially be true in military settings due to the intense nature of their work.

A significant challenge of wastewater monitoring faced by those without a background in microbiology is the ability to independently verify the accuracy of their results. Without training in the field, novices may not be able to identify their knowledge gaps. Solutions to possible issues related to data quality and other concerns with amateur data collection include—develop a data protocol that makes the work accessible to the participants involved, engage in active conversations with collaborators, have multiple in-person training sessions including touchpoints throughout the process, and incorporate third-party verification into the results collected (32–36).

## 4 Preparations for the next pandemic (or outbreak?)

Predicting the next possible infectious disease depends on factors such as the population to be monitored and disease transmission pathways. According to The National Academies of Sciences, Engineering, and Medicine, three key points need to be addressed when deciding on an approach for public health preparedness within the military (37). We have modified these into questions for military applications.

A. What is the risk of this infectious disease to the military?
B. How easy is it to detect the disease within samples collected by military personnel?
C. How does the data benefit the military for public health interventions?

Beyond the human pathogen targets currently being explored, another possible consideration would be the development of biological agents used in potential military attacks (biowarfare). Wastewater surveillance could be employed to detect viruses and other pathogens outside of what is currently known to maintain biosecurity. Some strategies to consider would include metagenomic screening, particularly for zoonotic agents outside of human sources. Further research on detecting emerging pathogens and antibiotic-resistant bacteria could benefit military applications by informing strategies to best protect deployed forces from biowarfare or pathogenic outbreaks (38). Yet the use of any technology should be coupled with resources that are attainable to the skill level of military personnel. While it is possible for one to automate or outsource the work to third parties, it would be beneficial if the laboratory processing and analysis could be accomplished by active duty non-expert personnel. It is also important to consider that there are perhaps different pathogens for different deployment sites.

## 5 Ethical considerations in WBE for military applications

The ethical challenges associated with wastewater surveillance have been well-documented, and briefly include data sensitivity, stigmatizing diseases, and misuse of data (19, 37, 39–46). This discussion highlights three important concerns. First, how should wastewater from decentralized systems (like septic tanks) be handled when it enters the public wastewater stream? Second, what measures can be imposed on a community during an active outbreak (39)? Third, how can individual privacy be protected through responsible data handling and reporting (41, 42)? The solutions suggested have included the incorporation of guidance from the World Health Organization (42, 45) and the active participation of affected stakeholders to participate in the monitoring program. While this may hold true for public wastewater systems, there would be a significant difference in application within the military. The military operates in a closed environment with commanders overseeing personnel (5). This suggests that the privacy concern pertains to the protection of both military and individual wastewater data. And so, possible ethics questions germane to the military could be as follows—

1. What is the chain of command engaged in the decision-making on monitoring, wastewater data handling/result production, and mitigation strategies?
2. Will superiors want to know the outcomes from a monitoring program? There may be some resistance to knowing and having to act on the results.
3. In the event of a viral outbreak on an active vessel, how would authorities balance the need for available deployed forces with the implementation of preventative measures to mitigate the spread of infectious diseases?
4. Will each military branch need to have its own separate wastewater monitoring protocol? Wastewater surveillance programs can help address this question.

Given these concerns, we propose a few recommendations to ensure ethical considerations in military wastewater surveillance programs. When establishing a surveillance program like this on a vessel, conducting a table top exercise that simulates a positive detection for a specific disease should be practiced. An exercise of this nature would include all parties involved in the response and would review necessary resources, privacy considerations, and actionable responses. Additionally, it is imperative to develop a communication chain in which if a positive detection is identified, what the following steps would include. This would likely require lab validation to ensure the detection is not a false positive, followed by a sequence of contacts to ensure that the information is provided to the appropriate individuals in a timely manner, so that any response measures could be quickly implemented. Data privacy and confidentiality measures that are employed in established wastewater surveillance programs (e.g., the Centers for Disease Control National Wastewater Surveillance System) could be easily transferred over to military-operated wastewater surveillance. Dissemination of disease findings and trends should not be kept in isolation though, and must be shared with crew members in a timely fashion (e.g., weekly). Crew members should all be notified of the program before embarking on the vessel, such as through a consent form, and should be alerted of disease trends to ensure that self-protective measures could be practiced if desired. While these are recommendations, there will still be opportunities to adapt and revise procedures, given that a program of this kind has never been implemented in a military setting. For example, identifying the conditions by which preference for viral outbreak mitigation should supersede military readiness will need to consider a multitude of factors that essentially evaluate the health risks for the deployed forces. Ultimately, numerous ethical considerations must be considered pertaining to privacy, response, and safety, but given the thousands of wastewater surveillance programs already implemented internationally, there are several frameworks that can be adapted to this unique setting.

## 6 Conclusion

This is the current state of wastewater surveillance within the context of military applications. Previously published studies (5–9) and a review of work published in confined civilian populations (15–31) provide a perspective of what might need to be considered if wastewater surveillance could be implemented in military branches. In summary, while wastewater surveillance studies have been conducted on military bases and training centers, research on active military vessels is lacking. However, studies of similar confined populations, such as those in colleges and universities, correctional facilities, and LMICs, could inform the development of an effective monitoring program for military vehicles. Furthermore, available technologies can simplify wastewater monitoring and data interpretation, enabling participation by military personnel regardless of their microbiological expertise. Our piece is possibly one of the few publications that have discussed these possibilities. We have found that WBE may be made possible in military applications if best practices are identified. Feasibility studies will better assess the protocols for wastewater surveillance in these environments.

## Data Availability

The original contributions presented in the study are included in the article/Supplementary Material, further inquiries can be directed to the corresponding author.
